# Whole-body DWI in patients with lymphoma: imaging findings, pitfalls, and limitations

**DOI:** 10.1186/1470-7330-14-S1-O22

**Published:** 2014-10-09

**Authors:** Alain Rahmouni, Sarah Toledano, Chieh Lin, Emmanuel Itti, Corinne Haioun, Alain Luciani

**Affiliations:** 1 Departement d’Imagerie Médicale, AP-HP, CHU Henri Mondor, Faculte de Medecine, Universite Paris-Est Creteil, France; 2 Department of Nuclear Medicine and Molecular Imaging Center, Chan Gung Memorial Hospital and Chang Gung University College of Medicine, Taoyuan, Taiwan; 3 Departement de Medecine Nucleaire, AP-HP, CHU Henri Mondor, Faculte de Medecine, Universite Paris-Est Creteil, France; 4 Departement d’Hematologie Clinique, AP-HP, CHU Henri Mondor, Faculte de Medecine, Universite Paris-Est Creteil, France; 5 TBC

## 

Whole body (WB) imaging plays an essential role in the management of lymphoma patients, including defining the full extent of the disease at baseline, allowing for an accurate staging and therefore an adapted treatment strategy, assessing treatment response and detecting relapse. Contrast-enhanced computed tomography (CT) has long been the imaging technique most commonly used for staging and follow up of malignant lymphoma, using International Working Group (IWG) criteria [1]. However, CT lacks functional and metabolic information, compromising identification of disease in non-enlarged lymph nodes or other organs, as well as sufficient contrast in certain organs as for example the spleen or bone marrow. In 2007, IWG response criteria were revised, incorporating Positron Emission Tomography (PET) with 18-fluorodeoxyglucose (FDG) information [2], thus combining metabolic information and anatomical data of the CT resulting in a higher accuracy than the both imaging modalities taken separately [3].

Diffusion Weighted Magnetic Resonance Imaging (DW-MRI) probes noninvasively the random microscopic motion of water molecules in the body, reflecting cellularity and cell membrane integrity. Because of their high cellularity and high nuclear-to-cytoplasm ratio, lymphomas have a lower apparent diffusion coefficient (ADC) than other tumors [4]. WB-DW-MRI allows both anatomical information, as well as functional and quantitative evaluation of tumor sites, thanks to the extraction of the apparent diffusion coefficient (ADC). At staging, lymphoma lesions have low ADC value except necrotic areas.

Recent studies comparing whole-body DWI to PET-CT have demonstrated the potential role of whole-body DWI in routine lymphoma patient care but included only a small number of patients. Using the DWIBS technique (diffusion weighted imaging with background body signal suppression), Abdulqadhr et al. compared whole-body DWI and PET/CT at staging with an agreement in the Ann Arbor stage for 90.3% of patients [5]. Based on ADC analysis, Lin et al showed an agreement at baseline in 93% of patients [4]. Response assessment is necessary during therapy to readapt treatment strategy if necessary, and to document a complete remission at the end of treatment. After treatment, an increase in ADC value of residual masses has been demonstrated [6]. Recent technical breakthroughs in MRI technology such as echo-planar imaging (EPI), high gradient amplitudes, combined phased-array surface coils covering the patient, and parallel imaging, have drastically improved patient comfort and acceptance for whole body MRI [7,8], making this technique feasible in clinical routine, illustrating the need for radiologists to get familiar with this technique. As a result, WB-DW-MRI with ADC mapping has become a promising tool for lymphoma staging and re-staging, and response assessment.

Based on our 4-years experience with WB-DW-MRI applied in Hodgkin and diffuse large B-cell lymphoma patients together with 18FDG-PET/CT, our objective is to offer radiologists the information required to optimize acquisition whole body DWI parameters on both 1.5 and 3T MR systems. We will expose the spectrum of imaging findings and discuss the pitfalls, limitations, and potential challenges of WB-DW-MRI in caring for lymphoma patients.
